# Characterization and Pharmacokinetic Assessment of a New Berberine Formulation with Enhanced Absorption In Vitro and in Human Volunteers

**DOI:** 10.3390/pharmaceutics15112567

**Published:** 2023-11-01

**Authors:** Julia Solnier, Yiming Zhang, Yun Chai Kuo, Min Du, Kyle Roh, Roland Gahler, Simon Wood, Chuck Chang

**Affiliations:** 1 ISURA, Burnaby, BC V3N 4S9, Canada; yzhang@isura.ca (Y.Z.); rkuo@isura.ca (Y.C.K.); mdu@isura.ca (M.D.); kroh@isura.ca (K.R.); cchang@isura.ca (C.C.); 2 Factors Group R & D, Burnaby, BC V3N 4S9, Canada; rgahler@factorsgroup.com; 3 School of Public Health, Faculty of Health Sciences, Curtin University, Perth, WA 6845, Australia; simonwood@shaw.ca; 4 InovoBiologic Inc., Calgary, AB Y2N 4Y7, Canada; 5 Food, Nutrition and Health Program, University of British Columbia, Vancouver, BC V6T 1Z4, Canada

**Keywords:** berberine, bioavailability, Caco-2 cell permeability, food-grade delivery system, LipoMicel, pharmacokinetics, solubility

## Abstract

Berberine is a plant-origin quaternary isoquinoline alkaloid with a vast array of biological activities, including antioxidant and blood-glucose- and blood-lipid-lowering effects. However, its therapeutic potential is largely limited by its poor oral bioavailability. The aim of this study was to investigate the in vitro solubility and Caco-2 cell permeability followed by pharmacokinetic profiling in healthy volunteers of a new food-grade berberine delivery system (i.e., Berberine LipoMicel^®^). X-ray diffractometry (XRD), in vitro solubility, and Caco-2 cell permeability indicated higher bioavailability of LipoMicel Berberine (LMB) compared to the standard formulation. Increased aqueous solubility (up to 1.4-fold), as well as improved Caco-2 cell permeability of LMB (7.18 × 10^−5^ ± 7.89 × 10^−6^ cm/s), were observed when compared to standard/unformulated berberine (4.93 × 10^−6^ ± 4.28 × 10^−7^ cm/s). Demonstrating better uptake, LMB achieved significant increases in AUC_0–24_ and C_max_ compared to the standard formulation (AUC: 78.2 ± 14.4 ng h/mL vs. 13.4 ± 1.97 ng h/mL, respectively; *p* < 0.05; C_max_: 15.8 ± 2.6 ng/mL vs. 1.67 ± 0.41 ng/mL) in a pilot study of healthy volunteers (*n* = 10). No adverse reactions were reported during the study period. In conclusion, LMB presents a highly bioavailable formula with superior absorption (up to six-fold) compared to standard berberine formulation and may, therefore, have the potential to improve the therapeutic efficacy of berberine. The study has been registered on ClinicalTrials.gov with Identifier NCT05370261.

## 1. Introduction

**Figure 1:**
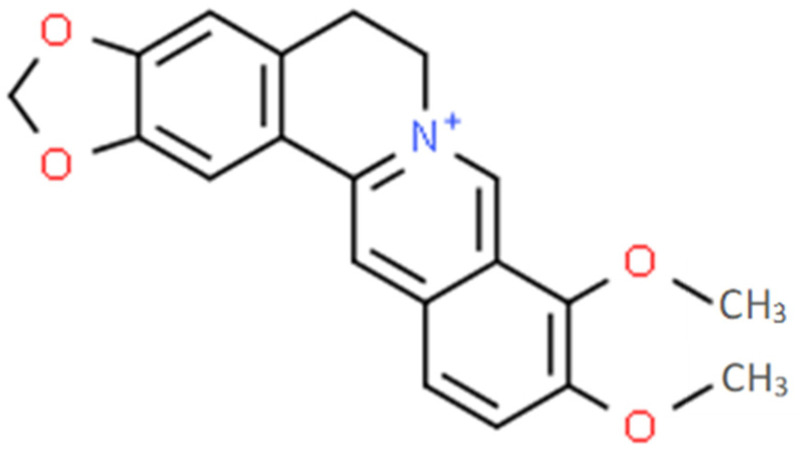
Chemical structure of berberine (Source: ChemSpider^®^ Database).

Berberine (2,3-methylenedioxy-9,10-dimethoxy-protoberberine) is a quaternary benzylisoquinoline alkaloid (C_20_H_18_NO_4_^+^; Figure 1) belonging to the class of protoberberine alkaloids. The alkaloid is extracted from different parts (e.g., root, rhizome, stem bark) of several plant species such as *Berberis vulgaris* (barberry), *Coptis chinensis* (Coptis or goldthread), and *Hydrastis canadensis* (goldenseal) [1].

In traditional Chinese and Indian medicines, berberine is commonly used to treat bacterial infections and inflammatory diseases [2], but it can also be effective in treating vascular and metabolic diseases such as atherosclerosis, hypertension, or diabetes [3].

In both experimental and clinical studies, berberine had beneficial effects on endothelial functions and improved glucose and lipid profiles [4]. Numerous studies have suggested berberine as a promising therapeutic agent to treat hyperlipidemia (e.g., in statin-intolerant patients [5,6]) and type 2 diabetes because it is effective at reducing blood lipid and glucose [7,8,9,10,11]. It also has a high tolerability profile [5,12] compared to conventional pharmaceutical drugs [13,14]. The lipid-lowering effects are primarily linked to the upregulation of hepatic LDL receptors (LDLRs) [8], the inhibition of proprotein convertase substilisin/kexin type 9 (PCSK9) [15], as well as the activation of AMP kinase (AMPK) [16]. The hypoglycemic effects of berberine—such as reducing fasting blood glucose and enhancing glucose uptake through stimulating insulin signaling pathways—are mainly associated with (a metformin-like) AMPK activation [17]. AMPK activation can also promote GLUT4 translocation, which indirectly accelerates the uptake of glucose and free fatty acids to the mitochondria by increasing ACC phosphorylation, both of which lead to a reduction in glucose and lipids [18]. Yin et al. reported that the hypoglycemic effect of berberine is similar to that of metformin at an equivalent dose of 1500 mg/day over a 3-month period, and cholesterol and triglycerides were lowered as well [7]. A systematic review and meta-analysis by Dong et al. concluded that the treatment of berberine together with other oral hypoglycemics significantly improved clinical outcomes related to fasting plasma glucose, glycosylated hemoglobin levels, fasting insulin levels, and triglyceride levels [19].

One major limitation of berberine’s clinical application is its low bioavailability. Orally administered berberine undergoes extensive metabolism (approx. 43.5% is metabolized in the enterocytes) due to p-glycoprotein (P-gp)-mediated efflux and self-aggregation, which greatly hinders its absorption [1].

The predominant form of berberine is the chloride (BCl) characterized by poor solubility in water (approximately 1.3 mg/mL [20]; classified as a class III molecule in the Biopharmaceutical Classification System), limited absorption rate, and extensive metabolic degradation within tissues, all of which present substantial obstacles in achieving effective delivery to specific target tissue sites [1,20,21,22].

In animal studies, the absolute bioavailability of berberine was found to be <1% (0.36% [23] and 0.68% [24]) after oral administration in rats due to its low absorption rate in the gut (approx. 33.6%) [25]. Tan et al. found considerably higher concentrations in the organs, such as the liver (e.g., 10-fold higher AUC) than in the plasma after orally administered berberine (200 mg/kg) [26]. In human pharmacokinetic studies, 5% or less of orally administered berberine was reported to enter the systemic circulation [2,27,28].

In both humans and rats, the compound is rapidly distributed and filtered out of the circulation by the liver, resulting in low systemic circulation levels of berberine [2]; it undergoes extensive hepatic metabolism involving phase I demethylation processes, followed by conjugation with sulfuric acid or glucuronic acid to produce phase II metabolites [29]. The higher accumulating concentrations of berberine in the tissues (i.e., liver, kidneys, muscle, lungs, brain, heart, pancreas) may explain its potent pharmacological effects observed in clinical studies (e.g., cholesterol and blood glucose reduction) at doses of 1000–1500 mg/day despite the low plasma concentrations after absorption [26,30,31,32].

Low oral bioavailability is commonly associated with the physicochemical properties (e.g., aqueous solubility, permeability, and stability in the GI tract) and the formulation of a compound (e.g., drug dispersion degree and dissolution), as well as certain physiological factors (e.g., efflux and extensive metabolism in the gut and liver) [23,33,34,35]. Therefore, several studies have explored different methods to improve the absorption of berberine in the body. One is the modifications of the chemical structure of berberine, such as long-chain alkylation (C_5_–C_9_) and 9-O-benzylation, that can lead to enhanced lipophilicity and bioavailability, as well as improved pharmacological efficacy [36,37]. Dihydroberberine, a derivative/metabolite of berberine, was found to produce significantly higher plasma concentrations of berberine than standard berberine [34]. Furthermore, numerous formulation techniques have been proposed to improve the uptake of berberine, which include, for example, nanostructured lipid carriers (NLCs) [38]; microspheres containing absorption enhancer (sodium N-[8-(2-hydroxybenzoyl) amino] caprylate (SNAC) [39]; as well as nano-formulations, including micelles [33,40,41] and liposomes [42,43]. Micro- or nano formulations are of special interest (e.g., polymeric-based, magnetic mesoporous silica-based, lipid-based, dendrimer-based, graphene-based, or silver and gold nanoparticles) that encapsulate a compound and provide a carrier for enhancing the intestinal uptake of molecules like berberine with low aqueous solubility [42,44,45,46]. Lipid-based delivery systems consisting of micro- or nanoparticles are characterized by their small particle size and high surface area-to-volume ratios [47], and have long been used in the pharmaceutical industry but recently also become prominent in the food and nutraceutical field [48].

This study investigated a new micellar lipid-based delivery system of berberine, namely LipoMicel^®^ Berberine (LMB), that disperses berberine into small microparticles together with food-grade ingredients to create a natural emulsion. Micelles are useful oral drug delivery systems for hydrophobic compounds due to their unique amphiphilic colloidal structure consisting of a hydrophobic inner core and a hydrophilic outer shell (core–shell) [49]. The key attributes of the current LipoMicel formulation are the small particle size (in the micrometer range), the lamellar micelle structure, lipid composition, entrapment efficiency and X-ray diffractometry (XRD) pattern, as well as the use of natural “food grade” ingredients. Compared to other studies using micellar formulations [33,40,50,51,52], LipoMicel contains natural and generally recognized as safe (GRAS) food ingredients without the use of synthetic detergents such as tweens/polysorbates, polyethylene glycols (PEGs), or poloxamers (e.g., pluronic F127). Although these chemicals are widely used in medical, pharmaceutical, cosmetic, industrial, and food products and are generally considered to be “safe” or “low toxic”, they have been associated with an increased prevalence of inflammatory bowel and metabolic diseases [53], and potentially can cause cross-reactivity with active compounds, leading to hypersensitivities or allergic reactions in rare cases [54,55,56,57].

The hypothesis of the current study was to determine whether berberine microencapsulated in a food-grade, lipid-based lipomicel formulation when compared to standard berberine treatment, would result in improved aqueous solubility and Caco-2 cell permeability in vitro, as well as improved pharmacokinetics (such as AUC_0–24_ and C_max_) in human volunteers, thereby increasing the oral bioavailability of berberine.

## 2. Materials and Methods

### 2.1. Participants

**Figure 2:**
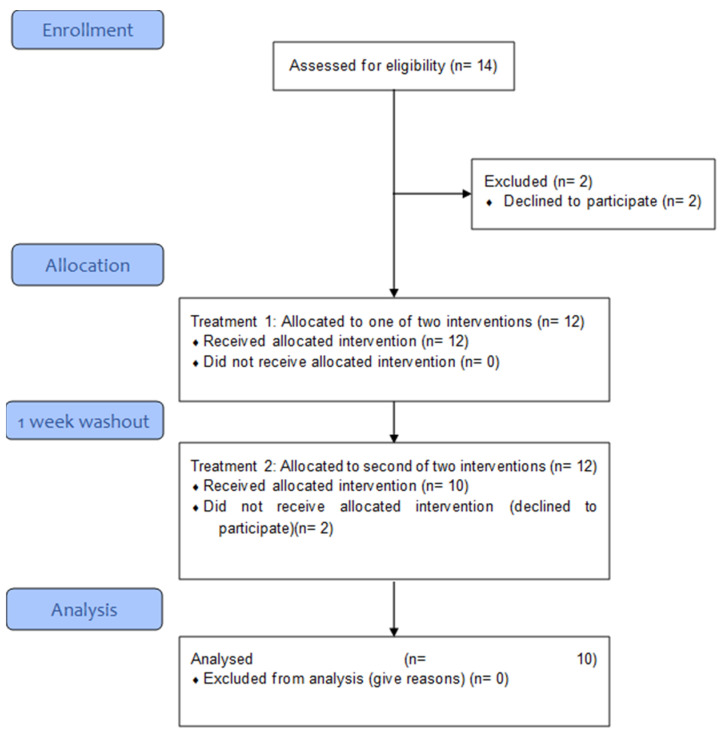
Study flow diagram.

Fourteen healthy adults were initially recruited for this study. Ten healthy adults of both sexes (5 men; 5 women; average age 34 years with a mean BMI of 22.3 ± 2.2 kg/m^2^ and mean weight of 63.3 ± 9.5 kg) completed the entire trial and were included in the analysis (Figure 2). Healthy participants who were ≥21 years old, of good physical condition—non-smokers, not taking any prescribed medication—were included in the trial.

All participants provided their written informed consent before participating in this study. Forty-eight hours before each treatment and during the treatment period, participants were instructed to refrain from taking supplements containing berberine and to maintain a normal, balanced diet.

The inclusion criteria included a signed written informed consent form and a willingness to avoid the consumption of any herbal supplements that contain berberine (48 h before each treatment and during the respective treatment periods). As exclusion criteria, participants must not have any of the following diseases and/or health conditions: serious acute or chronic diseases—such as liver, kidney, or gastrointestinal diseases—which may affect absorption, metabolism, and/or elimination of the treatment, as well as any kind of contraindication and/or allergy to berberine. Female participants must not have been pregnant, planning to get pregnant, or be breast-feeding. Participants had to complete an online health questionnaire on their medical history upon study enrolment.

### 2.2. Treatments

In this study, different formulations of berberine were purchased from commercial sources and investigated (Table 1). Each product in this study was administered (one or two capsules) as a single dose containing a total dose of 500 mg of berberine:

- Standard berberine formulation (WellBetX^®^; WBX) was purchased from Natural Factors, BC, Canada. One hard-gelatin capsule contained 500 mg of berberine hydrochloride from *Berberis vulgaris* root.
- A new food-grade berberine delivery system (LipoMicel^®^ Berberine; LMB) was provided by Natural Factors, BC, Canada. One soft-gel capsule contained 250 mg of berberine from *Berberis vulgaris* root with medium-chain triglycerides and food-grade components of the micellular membrane with food-grade excipients (patent pending—LipoMicel^®^ Matrix).

### 2.3. Safety and Tolerability

Potential adverse events associated with the study treatments were evaluated through the collection of health questionnaires.

### 2.4. Pharmacokinetic Study in Healthy Volunteers

A diet-controlled, blinded, crossover pharmacokinetic study was performed with commercial berberine formulations. The study was approved by the Canadian SHIELD Ethics Review Board (OHRP Registration IORG0003491; FDA Registration IRB00004157; Approval letter ID 2021-08-004, date of approval: 24 August 2021). The study has been registered on ClinicalTrials.gov with Identifier NCT05370261 and conducted in accordance with the ethical standards as set forth in the Helsinki Declaration of 1975.

On the day of the trials, capillary blood samples were taken after a 10 h overnight fast to determine the initial berberine concentration (baseline: t = 0). Thereafter, each participant received an oral dose of either 1 capsule of 500 mg WBX or 2 capsules of 250 mg LMB and continued to fast for 4 h. While participants were aware that two different formulations with different bioavailabilities were being tested, they did not know the name or composition of the products.

Following the interventions, capillary whole-blood samples were collected at time points 0.5, 1, 2, 3, 4, 6, 8, 10, 12, and 24 h after the single-dose administration of berberine. Standardized lunch and dinner were served after 4 and 8 h of product administration, respectively. Coffee- or tea-based beverages were allowed after a 2 h fasting. Water could be consumed ad libitum during each study session.

At each blood sampling time point, capillary blood samples (50 µL) were drawn into microcentrifuge tubes. The vials were closed immediately and kept frozen at −20 °C until further processing and analysis. Processed samples were loaded into a refrigerated autosampler to be analyzed by LC-HRMS within 24 h after processing. Each experimental session was conducted at the lab facility of ISURA (Burnaby, BC, Canada).

The Interventions were conducted in a crossover fashion with a minimum washout of 7 days (Figure 2).

#### Blood Sample Preparation and Analytical Procedures

Sample preparation and analytical protocols were adapted from a previously published method [58]. The collected whole-blood samples were first thawed at room temperature, and then 50 µL of blood was treated with 100 µL of β-glucuronidase (from *Helix pomatia*, ≥100,000 IU diluted to 330 IU; Millipore-Sigma, Burlington, MA, USA) in pH 5 buffer and incubated for one hour at 37 °C. Berberine chloride traceable to a certified reference material (Millipore-Sigma, Burlington, MA, USA) was used for the stock solutions for the calibration standards and quality controls. Benzanilide (Millipore-Sigma, Burlington, MA, USA) was included as an internal standard before sample analysis, as previously published [58,59]. In total, 400 µL of methanol (ACS grade, Fisher Chemical, Toronto, ON, Canada) was added to extract the samples. Samples were sonicated for 15 min while maintained in a water bath at room temperature. After extraction, tubes were centrifuged at 16,000× *g* for 5 min at 25 °C. The supernatant was transferred into a microplate for LC-HRMS analysis. Processed samples were analyzed using a Vanquish Ultra High-Performance Liquid Chromatography (UHPLC) system (Thermo Fisher Scientific Inc., Waltham, MA, USA) coupled to a Q Exactive^TM^ Orbitrap^TM^ Mass Spectrometer (Thermo Fisher Scientific Inc., Waltham, MA, USA). Briefly, 20 µL of each sample was injected into the instrument with a binary solvent gradient progressing from 30% B to 75% B in 4 min and equilibrated for 5 min before the next injection. The mobile phases were 0.5% formic acid in water in A and methanol in B. An Acme Xceed C18, 100 mm × 2.1 mm, 1.9 µm UHPLC column (Phase Analytical Technology, State College, PA, USA) was used to perform the separation at a flow rate of 400 µL/min.

The Orbitrap mass spectrometer was calibrated at 70,000 resolution with an accepted range for mass deviation of +/−5.0 ppm using Pierce^TM^ LTQ Velos ESI Positive-Ion Calibration Solution (Thermo Fisher Scientific Inc., Waltham, MA, USA) and Pierce^TM^ ESI Negative-Ion Calibration Solution (Thermo Fisher Scientific Inc., Waltham, MA, USA). To reduce interference from the sample matrix, the mass spectrometer was operated in timed Selected Ion Monitoring (tSIM) Mode with heated electrospray at a resolution of 70,000 and a quadrupole isolation window of 3.0 m/z. Benzanilide (internal standard) was detected as a hydrogen adduct with a mass of 198.0913. Berberine was detected as a hydrogen adduct with a mass of 336.1230 and as berberine formate with a mass of 382.1285.

Data were collected using Xcalibur^TM^ 5.0 (Thermo Fisher Scientific Inc., Waltham, MA, USA) and analyzed with TraceFinder 5.0 (Thermo Fisher Scientific Inc., Waltham, MA, USA) software with the default mass tolerance set to 5.00 ppm. Concentrations of berberine in capillary whole blood were determined based on internal standard calibration with a 6-point calibration curve using berberine hydrochloride as the chemical standard (Certified Reference Material, secondary standard, Millipore Sigma, Burlington, MA, USA).

This method was validated according to the European Medicines Agency’s ICH Guideline M10 on bioanalytical method validation for selectivity, specificity, calibration curve and range, accuracy and precision, and carryover. The calibration concentrations were selected based on ICH M10 and ranged from 1 to 100 ng/mL. Quality controls were evaluated at 4 concentrations (1, 3, 10, and 80 ng/mL) to determine accuracy and range.

**Figure 3:**
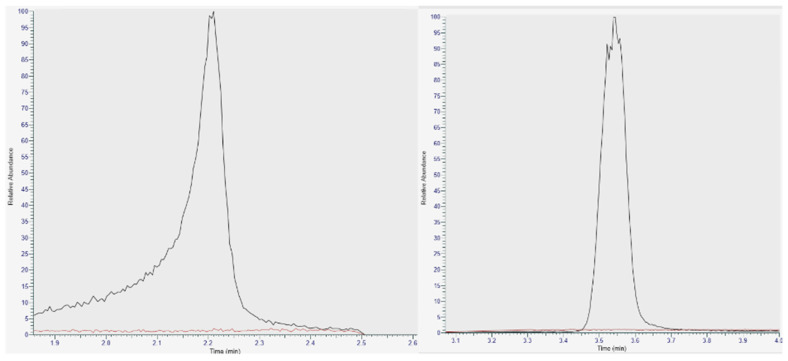
LC-HRMS overlay chromatogram of berberine; (**left**) berberine at 1 ng/mL; (**right**) benzanilide at 50 ng/mL. Black trace—berberine in human blood. Red trace—matrix blank. Raw chromatograms are presented without any signal-processing algorithms applied so that more signal noise and chromatographic distortions would be observed.

Selective and specificity are demonstrated by the overlay chromatograms (Figure 3). Calibration curve linearity has an average R2 value of 0.999. Average precision is 10%, average accuracy is 5.1% of nominal value, and carryover is 0.76%.

### 2.5. X-ray Diffraction

X-ray diffraction (XRD) patterns of berberine products were acquired with a Rigaku MiniFlex 600 6G diffractometer using Cu Kα radiation (λ = 0.15418 nm) and a 2D HyPix −400 MF detector operating in one-dimension mode (4D LABS, Simon Fraser University, Burnaby, BC, Canada). Copper X-rays were generated from a copper target by electron bombardment at 40 kV and 15 mA. The incident and receiving Soller slits were 5 degrees. A 0.625-degree divergence slit was placed between the X-ray source and the sample. An 8 mm scatter slit and a Ni filter, to diminish Cu K-beta radiation, were placed between the sample and the detector. Samples were mounted on a glass sample holder.

### 2.6. Particle Size Distribution

The Mastersizer 3000 particle size analyzer (Malvern Panalytical, Québec City, QC, Canada) was used to determine the particle size distribution. Briefly, approximately 1 mL or 1 g of the content of the capsules was added into a Hydro SM (Malvern Panalytical, Québec City, QC, Canada) wet dispersion accessory filled with approximately 200 mL of water. Data were collected over a period of 1 min once the dispersed mixture reached 10% obscuration. Hydrodynamic volumes of the resulting particulates (powder-in-water and micelle-in-water mixtures) were determined through laser diffraction data analyzed using the Mastersizer software 3000 (Malvern Panalytical, Québec City, QC, Canada).

### 2.7. Cryo-SEM

Around 400 mg LMB soft-gel fill material was dispersed in deionized water to make 1.5 mL suspension in a 1.5 mL polypropylene microcentrifuge tube with snap cap. The suspension was sonicated in a warm bath (30–40 °C) for 15 min and then allowed to settle for 5 min. A few drops of light-colored top portion of the suspension were filled into wells made on an aluminum cryo-SEM holder with a small amount of overfill. The cryo-SEM holder with the sample was then submerged in a slushy liquid nitrogen for 10–20 s to rapidly freeze the samples. After freezing, the sample was vacuum transferred into a Quorum PP3010T cryochamber (Quorum Technologies, East Sussex, UK) to fracture the overfill portion off in order to reveal the cross-section of the frozen sample. The fractured sample was then further transferred in a Helios NanoLab 650 scanning electron microscope (FEI Company, Hillsboro, OR, USA) for imaging. Cryo-SEM images were collected with a current of 13 pA at 2 kV, with a working distance of 4 mm, at a scanning resolution of 3072 × 2207 or lower by averaging 128 low-dose scanning frames with drift correction. The sample was kept at −140 °C when fracturing and imaging. The sample was also imaged after sublimation at −80 °C for 15 min in a cryo-SEM chamber to remove some water.

### 2.8. Solubility

The berberine formulations used in this study were analyzed in terms of their solubility in distilled water and simulated gastric and intestinal solutions. Simulated gastric and intestinal solutions were prepared according to the method published by USP. An excess amount of the berberine sample was added with 10 mL of liquid in a 15 mL centrifuge tube to reach saturation.

Samples were vortexed briefly to suspend visible particles and then sonicated for 15 min at 37 °C to ensure saturation of the aqueous phase of the mixture. Next, particles and micelles were removed by filtering through 0.45 μm polytetrafluoroethylene (PTFE) filters (Chromatographic Specialties, Brockville, ON, Canada), and particulate-free filtrates were transferred into glass vials for the quantification of berberine using a high-performance liquid chromatography (HPLC) system. Filtered samples contained only particles smaller than 0.45 μm. Filtered samples were analyzed using a Ultimate 3000 RS UHPLC system (Thermo Fisher Scientific Inc., Waltham, MA, USA) with a quaternary pump delivering a binary gradient of 0.2% phosphoric acid (HPLC grade, VWR International, Mississauga, ON, Canada) in HPLC-grade water (Fisher Scientific, Toronto, ON, Canada) and HPLC-grade acetonitrile (Fisher Scientific, Toronto, ON, Canada) through a Poroshell EC-18 100 × 2.1 mm, 2.7 μm column (Agilent Technologies, Santa Clara, CA, USA) at 0.600 mL/min. Gradient was linearly increased from 12% acetonitrile to 95% acetonitrile over a period of 12 min. The column was equilibrated with the starting conditions for 2 min before the next injection. The column oven was set to 40 °C, and data were collected at 235 nm.

### 2.9. In Vitro Caco-2 Cell Permeability Studies

Caco-2 cells (Cedarlane Laboratories, Burlington, ON, Canada) were cultured in a T-25 flask (Thermo Fisher Scientific Inc., Waltham, MA, USA) in a HERACELL VIOS 160i CO_2_ incubator (Thermo Fisher Scientific Inc., Waltham, MA, USA) set to 37 °C and 5.0% CO_2_. The composition of the cell culture media was as follows: Dulbecco’s modified Eagle’s medium (DMEM) (Sigma-Aldrich, Burlington, MO, USA), 10% heat-inactivated fetal bovine serum (FBS) (Thermo Fisher Scientific Inc., Waltham, MA, USA), penicillin (100 units/mL), and streptomycin (100 units/mL) (Sigma-Aldrich, Burlington, MO, USA). For the permeability study, cells were resuspended with 5% trypsin (Thermo Fisher Scientific Inc., Waltham, MA, USA) and seeded on a 24-well culturing plate with a format polycarbonate semipermeable membrane insert (6.5 mm diameter, 0.4 μm pore size; VWR International, ON, Canada). The seeding density was 1 × 10^−4^ cells/cm^2^. Seeded cells were incubated with the culture media for a total of 21 days before the permeability assay. The culture media were refreshed every 48 h during the first 14 days, and then every 24 h prior to the test. An EVOM2 instrument (World Precision Instruments, Sarasota, FL, USA) was used to measure the transepithelial electrical resistance (TEER) values of the cells. Only Caco-2 monolayers with TEER values between 250 and 500 Ωcm^2^ were selected for use in permeability experiments.

On the day of measurement, Caco-2 cells were washed twice with Hanks’ balanced salt solution (HBSS) (Sigma-Aldrich, Burlington, MO, USA) and then allowed to equilibrate for 30 min in the incubator at 37 °C. Next, Berberine formulations were diluted 1 in 10 in culture media, centrifuged at 10,000× *g* for 3 min to remove particulates, and then the supernatants were added separately as donor solutions to the apical side of the monolayer, and 500 µL of HBSS solution was added to the basal side. Four hours after the treatment, the basal solution was collected for LCMS analysis. All treatments were performed in triplicate. A control sample consisting of only LMB excipients was also tested, and no significant changes in TEER values were observed.

The apparent permeability coefficient (*P_app_*) can be calculated from the permeation rate and compound concentration at t = 4 h (see formula below). In this formula, *dQ*/*dt* is the amount of product present in the basal compartment as a function of time (nmol/s), A is the area of transwell (cm^2^), and *C*_0_ is the initial concentration of product applied in the apical compartment (μM).

$$P_{app}\;=\;\frac{dQ}{dt}\cdot \frac{1}{A\cdot C_{0}}$$

### 2.10. Data Analysis

The following pharmacokinetic (PK) parameters were evaluated: the time to reach peak blood concentration (T_max_), maximum total berberine blood concentration (C_max_), the elimination rate constant (K_el_), and the area under the total berberine blood concentration curve from 0hr (administration time) to 24 h (AUC_0–24_). A noncompartmental pharmacokinetic analysis (NCA) was conducted to calculate the PK parameters using sampling times by means of the software PCModFit V.6.7 (add-on for Microsoft Excel).

As for statistical analysis, comparison of the different pharmacokinetic parameters between the two treatment groups was performed using ANOVA (Mixed Model) repeated measures with the Šidák multiple comparisons test. Differences in solubility and caco-2 cell permeability between the two treatments were evaluated using unpaired *t*-tests.

Prior to the statistical tests, normality was assessed using the Shapiro–Wilk test. Data were considered significant at *p* < 0.05. GraphPad Prism software ver. 10.0.3 (GraphPad Software Inc., La Jolla, MA, USA) was used for the statistical analyses and graphical presentation.

## 3. Results

### 3.1. Pharmacokinetics of Different Berberine Formulations

**Figure 4:**
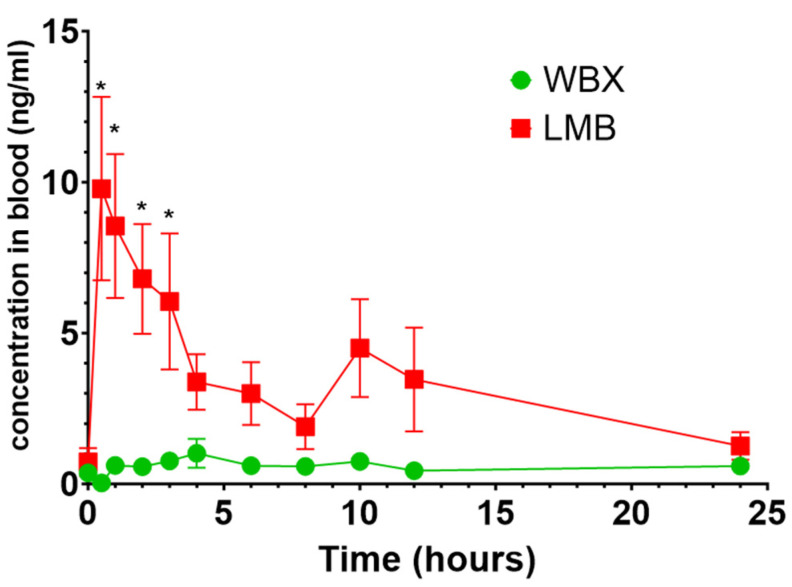
Comparison of pharmacokinetic profile among the two berberine formulations. Average blood concentration of berberine LipoMicel (LMB; red) and standard berberine WellbetX (WBX; green). Data presented as mean ± SEM, *n* = 10 for each group; * *p* < 0.05 (ANOVA).

The absorption of two different berberine products was monitored over a 24 h period in 10 healthy participants following the oral administration of 500 mg berberine per treatment. Blood concentrations of berberine are presented in Figure 4 and Table 2.

LMB achieved significantly higher absorption when compared to unformulated/standard berberine (AUC: 78.2 ± 14.4 ng h/mL vs. 13.4 ± 1.97 ng h/mL; *p* < 0.05, respectively). Furthermore, LMB attained up to 10-fold higher peak concentrations (C_max_) during the 24 h period (C_max_ = 15.8 ± 2.6 ng/mL) than WBX (1.67 ± 0.41 ng/mL). The time to reach maximum/peak concentrations was approx. five times shorter with LMB compared to WBX (T_max_: 1.06 ± 0.21 h vs. 5.60 ± 0.94 h), indicating the faster absorption of LMB. Although not significant, LMB showed a lower elimination rate constant (K_el_) compared to standard berberine treatment, indicating a slower elimination of LMB, resulting in higher blood concentrations. A previous study on standard berberine reported a similar elimination half-life of 2.94 ± 0.14 h, which is equivalent to an elimination rate constant of 0.236 h^−1^. [60]

**Figure 5:**
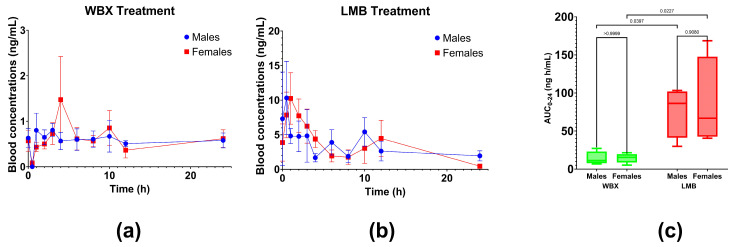
Berberine absorption of individual treatments in female (*n* = 5) vs. male participants (*n* = 5). Data are sub-grouped by gender; blood concentrations for each gender are compared over time for (**a**) WBX: WellbetX (standard berberine) and (**b**) LMB: LipoMicel with no significant differences observed between genders (*p* > 0.05; ANOVA). (**c**) Comparison of AUC_0–24_ values between genders for the two treatments (WBX: green and LMB: red).

Figure 5 and Table 3 demonstrate the gender differences in berberine absorption in female (*n* = 5) and male (*n* = 5) participants within the individual treatment groups (LMB and WBX). The results revealed no significant differences in AUC berberine in male vs. female participants (WBX: *p >* 0.9999 and LMB: *p* = 0.9080; Figure 5), as well as in C_max_ and T_max_ (Table 3).

No side effects/adverse events during the study period were reported, indicating that the treatments were well tolerated.

### 3.2. Solubility of Different Berberine Formulations

The results of the in vitro solubility studies performed with different formulations of berberine are summarized in Table 4. LMB had up to 1.4-fold better solubility in water as well as significantly higher solubility in simulated gastric solution compared to WBX. However, when tested in simulated intestinal media at pH 6.8, both formulations had similar solubility.

### 3.3. Permeability of LMB vs. WBX in Caco-2 Cells

**Figure 6:**
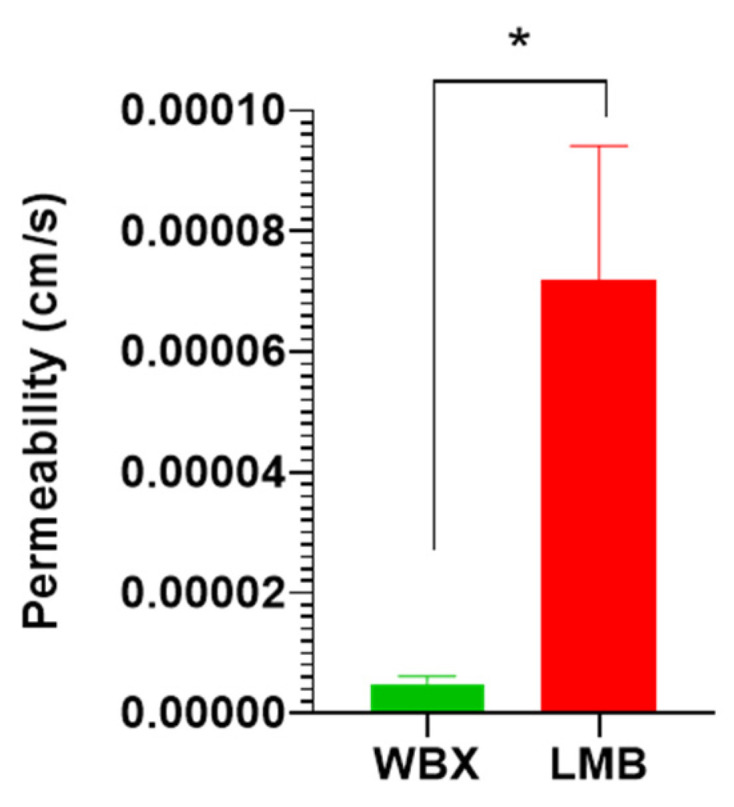
Caco-2 monolayer permeability with LMB: LipoMicel Berberine and WBX: WellBetX (standard berberine); *n* = 8; * *p* < 0.001 (unpaired *t*-test).

The apparent permeability coefficient (*P_app_*) of the compounds is a measure of the rate at which a compound can cross an area of the cell monolayer [61]. The results of the Caco-2 cell permeability studies performed with LMB and WBX indicated a significantly higher cell permeability of LMB (7.18 × 10^−5^ ± 7.89 × 10^−6^) compared to WBX (4.93 × 10^−6^ ± 4.28 × 10^−7^ cm/s), as summarized in Figure 6 and Table 5. These findings are consistent with the aqueous solubility results, highlighting greater permeability of LMB in human intestinal epithelial (Caco-2) cells. This is also confirmed by the significantly higher pharmacokinetics of LMB in study participants.

### 3.4. Cryo-SEM of LMB

**Figure 7:**
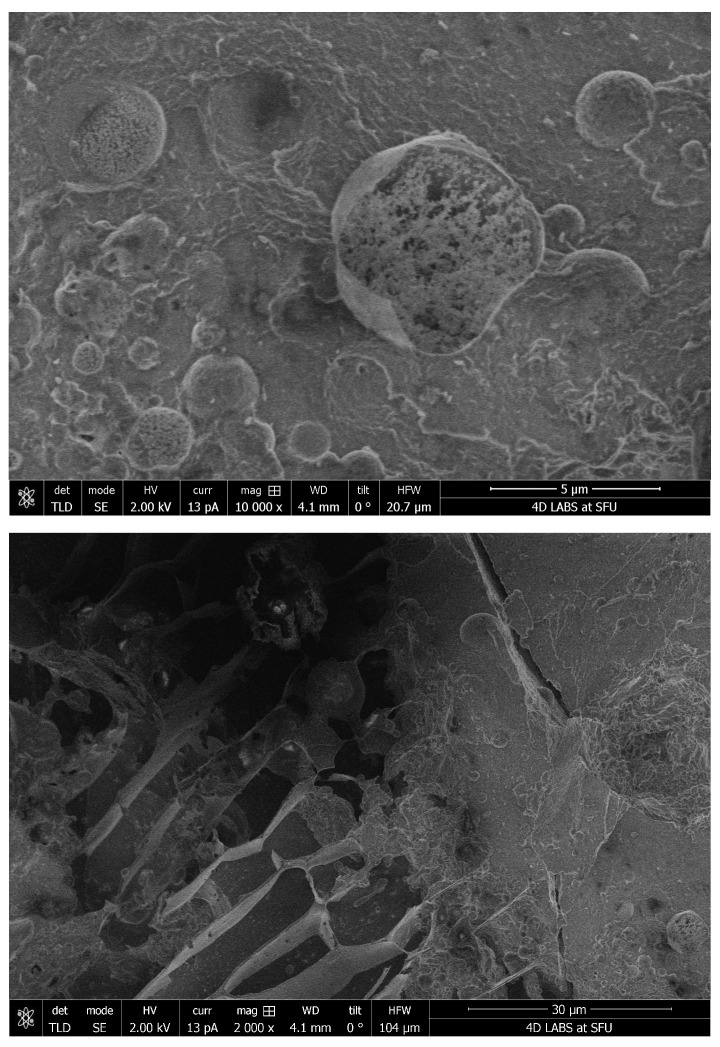
Cryo-SEM characterization. (**Top**): LMB (LipoMicel Berberine); (**Bottom**): WBX (WellBetX; standard berberine).

Cryo-SEM reveals that the berberine present inside the micelles of LMB is amorphous, minute, and irregular. This is in sharp contrast with WBX powder (Figure 7) which reveals large, smooth, flat surfaces characteristic of crystalline structures.

### 3.5. X-ray Diffraction of the Berberine Products

**Figure 8:**
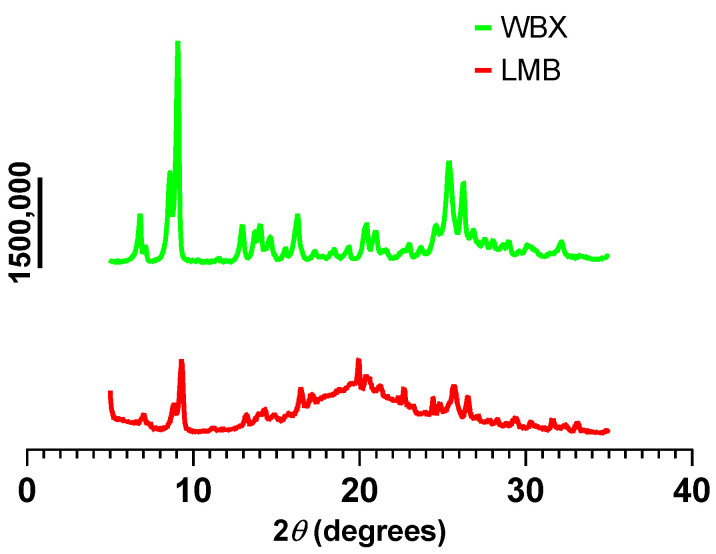
A comparison of X-ray diffraction between the berberine formulations. LMB: LipoMicel Berberine (red); WBX: WellBetX (standard berberine; green).

X-ray diffraction plots present the relative degree of crystallinity in the different berberine products (Figure 8). X-ray diffraction has been recognized as a reliable technique to study the mechanical properties and surface structure of crystals while providing insight into their bioavailability potentials [62]. When a beam of X-rays is directed at a crystalline material, the atoms in the crystal scatter the X-rays in all directions. However, because the atoms in a crystal are arranged in a regular, periodic pattern, the diffracted X-rays will also have a regular, periodic pattern. Sharper diffraction peaks indicate a higher degree of crystallinity, whereas diffraction peaks with less intensity are indicative of amorphous and less crystalline (semi-crystalline) materials [62,63]. As shown in Figure 8, the LMB (red) exhibits a nearly flat curve with small peaks. This is consistent with an amorphous phase and suggests better solubility along with the higher bioavailability of berberine. In contrast, the WBX product (green) presents diffraction patterns with a series of sharper peaks associated with highly crystalline materials and suggests less bioavailability of berberine.

### 3.6. Particle-Size Distribution by Laser Diffraction

**Figure 9:**
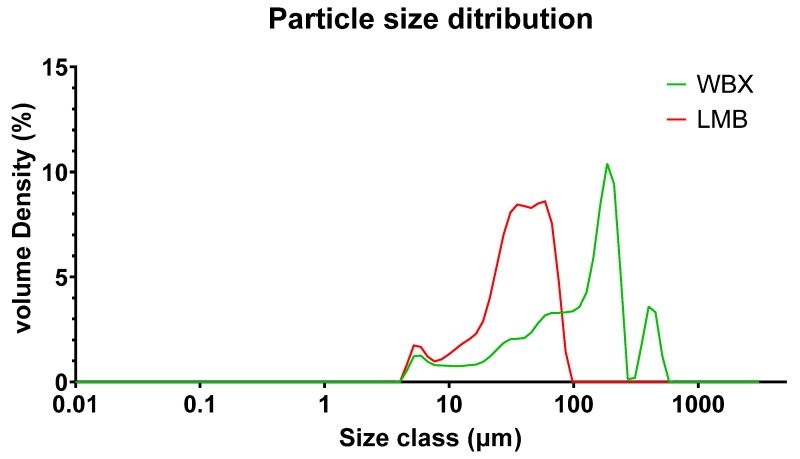
Particle size distribution determined by laser diffraction; *n* = 3; the average volume density plotted against the average particle size. Volume density is a measure of the proportion of particles present at each size interval based on their volume; the size classes represent the particle size in micrometers; LMB: LipoMicel Berberine (red); WBX: WellBetX (standard berberine; green).

Laser diffraction measurements are taken in triplicate for both formulations, and Figure 9 presents the average of the three datasets. The data represent the hydrodynamic volumes of the micelles in the case of LMB and the hydrodynamic volumes of the powder-in-water particulates in the case of WBX. LMB generates a smaller distribution profile than WBX, with a particle size ranging from a few micrometers to larger than 50 micrometers. This likely corresponds to its higher solubility in the tested media (i.e., water, the carrier fluid used in the Malvern analyzer).

## 4. Discussion

Previous studies on LipoMicel have highlighted its potential to improve the absorption and physicochemical properties of natural compounds [64,65].

In this work, both the in vitro and in vivo absorption characteristics of a new micelle formulation of berberine, named LipoMicel Berberine (LMB), have been evaluated. Solubility studies revealed that berberine microencapsulated as a LipoMicel matrix (i.e., LMB) results in higher aqueous solubility compared to standard berberine (approx. 142% of that of regular formulation (WBX)); this further corresponded to the XRD patterns of LMB showing diffraction peaks with less intensity and sharpness compared to WBX, which is indicative of an amorphous phase and a change in the crystalline nature of the material [63]. Less crystalline or more amorphous compounds have better solubility and greater Gibbs free energy [66], as well as enhanced dissolution rates compared to their respective crystalline forms and are, therefore, more bioavailable. As shown in previous studies, modifications in the crystalline nature, for example, through micro- or nano-sizing or by creating an amorphous solid dispersion like LMB, may be among the most effective approaches to enhance the solubility, dissolution, as well as the permeability of molecules, and consequently their bioavailability [62,63,67,68].

Caco-2 cells have been used to predict the in vivo oral absorption of berberine as they provide a model of the human intestinal epithelium, the first barrier to absorption and to reach the systemic circulation. Therefore, the transport of LMB and WBX through the Caco-2 cell monolayer was investigated to evaluate their ability to permeate the epithelium layer. The permeability results showed the significantly higher permeability of LMB in Caco-2 cells compared to WBX; the apparent permeability coefficient (*P_app_*) values reported in this study are in accordance with the findings of other studies on berberine compounds [69]. The enhanced intestinal permeability in Caco-2 cells is likely the result of both the small LipoMicel particles (size distribution in 5–100 micrometers range; Figure 9) and the natural surfactants, which may prevent p-glycoprotein-mediated efflux, as previously suggested by Kwon et al. [33].

In general, small particles such as micro- or nanoparticles are transported through the cell by endocytotic mechanisms such as pinocytosis, macropinocytosis, or clathrin-mediated endocytosis [70]. For instance, Qu et al. reported that the enhanced permeability of paclitaxel-loaded mixed polymeric micelles in Caco-2 cells was associated with both clathrin- and caveolae-mediated micropinocytosis mechanisms [71].

As shown in a previous study, the different compositions of natural emulsifiers (e.g., phosphatidylcholine lecithin) and biosurfactants (e.g., medium-chain triglyceride (MCT)) in a LipoMicel formulation can help to significantly increase the intestinal permeability in Caco-2 cells [65]. Park et al. reported that permeability-enhancing surfactants consisting of medium-chain fatty acids may penetrate the lipid bilayer more easily because of their proper lipid solubility [72]. Furthermore, natural low-molecular-weight surfactants, such as saponins and lecithins, have been proven to be effective at forming and stabilizing emulsions, thus improving the intestinal uptake [73].

To test if the improved aqueous solubility and Caco-2 cell permeability of LMB would result in higher blood concentrations, a pilot crossover study on healthy human volunteers was conducted. Artursson et al. demonstrated a clear correlation between the *P_app_* of several passively diffused drugs in the Caco-2 model and the absorbed fraction of these drugs after oral administration in humans [36]. In this work, the higher *P_app_* of LMB compared to WBX (7.18 × 10^−5^ vs. 4.93 × 10^−6^) was reflected in significantly higher blood concentrations (AUC 78.2 ± 14.4 vs. AUC 13.4 ± 1.97 ng h/mL; C_max_ 15.8 ± 2.6 vs. 1.67 ± 0.41 ng/mL) in the study participants. The time taken to reach the maximum plasma concentrations (T_max_) was significantly shorter with LMB (T_max_: 1.06 h) compared to standard berberine WBX (5.6 h). The discrepancy in T_max_ is likely related to the differences in the physicochemical and formulation factors of LMB and WBX (i.e., soft gel vs. hard gel), which can affect the dissolution properties and bioavailability. Compared to a hard-gel capsule (a solid dosage form), a soft-gel capsule contains a liquid, a suspension, or a semisolid material filled in a one-piece hermetically sealed shell. Soft gel forms can offer several advantages over traditional oral solid dosage forms [74]. For example, they can mask the unpleasant taste and odor of compounds and are more comfortable to swallow due to their self-lubricating nature when used with water [74]; they tend to have higher stability and protect compounds against oxidation, degradation, and contamination [74] and can enhance the bioavailability of poorly soluble compounds by using lipophilic vehicles as part of their fill material [75,76].

Only a few studies have reported the pharmacokinetics of berberine in humans. In a similar study, berberine Phytosome^®^ (also a food-grade delivery system) achieved up to 4-fold increases in total berberine concentrations (AUC 4952 ± 647 vs. 1217 ± 129  pg h/mL; Cmax 76.70 ± 14.04 vs. 316.88 ± 26.60 pg/mL) compared to unformulated berberine when tested at similar doses, as used in the current study [77]. Moon et al. found that dihydroberberine (DHB), the reduced derivative of berberine, leads to considerably higher berberine concentrations in their study participants than standard berberine at doses of 100 mg DHB vs. 500 mg berberine (AUC 284.4 ± 115.9 vs. 42.3 ± 17.6 ng h/mL; C_max_ 3.76 ± 1.4 vs. 0.4 ± 0.17 ng/mL, respectively) within a 2 h period. However, the increased bioavailability was not accompanied by better clinical efficacy since no significant changes in glucose and insulin response were observed during the study period [34]. Hua et al. reported a mean C_max_ and AUC_0–∞_ of approx. 0.4 ng/mL and 9.2 ng h/mL, respectively, in 20 study participants orally administered 400 mg of berberine [27]. Similar results were reported after the administration of 500 mg of berberine in 10 volunteers with a C_max_ value of 0.07 nM [78].

Based on the existing literature, it can be suggested that LMB achieves higher absorption through the following mechanisms: reduced particle size and improved aqueous solubility [42] and intestinal permeability through the endocytosis of encapsulated berberine across the intestinal epithelia; reduced intestinal first-pass elimination, as well as hepatobiliary re-excretion [23,79] and self-aggregation [80]; and reduced p-glycoprotein efflux in the intestine and the liver [35,81,82]. Several studies using a Caco-2 cell monolayer model have demonstrated that p-glycoprotein efflux considerably contributes to low concentrations of berberine in small intestinal epithelial cells [35,83]; thus, taking berberine along with p-glycoprotein inhibitors such as silymarin from Milk Thistle or d-α-tocopheryl polyethylene glycol 1000 succinate (TPGS) has been shown to increase absorption [24,84]. Since berberine is a known p-glycoprotein inhibitor [85], it is possible that LMB’s properties, such as increased solubility and reduced particle size, led to reduced intestinal p-glycoprotein efflux [86]. 

CYP1A2, CYP2D6, and CYP3A4 have been reported to be involved in the berberine metabolism, as well as the intestinal microbiota [25,87]. In humans, berberine was found to decrease CYP2D6, 2C9, and CYP3A4 activity [88], which may cause herb–drug interactions. Therefore, novel formulations such as LMB that increase the blood concentrations of berberine should be further investigated in terms of their effects on CYP activities, especially since berberine appears to be a good candidate for the adjunctive treatment of diabetes and patients with suboptimal glycemic control [89].

Interaction studies between the gut microbiota and orally administrated berberine are another important field of research [90]. Variations in the gut microbiota may contribute to inter-individual and intra-individual differences in drug metabolism [91]. For instance, Alolga et al. reported significant pharmacokinetic differences in berberine between African and Chinese study participants (AUC_0–12_: 0.96  ±  0.34 vs. 0.47  ±  0.13, respectively), likely due to variations in gut microbiota [54]. Since different factors can affect drug absorption (e.g., age, diet/microbiome, ethnicity, health status, sex differences, etc. [92]), in this study, the pharmacokinetic differences between female and male participants were investigated; however, no significant differences in AUC, as well as C_max_ and T_max_, were observed.

In the current study, LMB demonstrated higher intestinal permeability through a Caco-2 cell monolayer and higher absorption in human study participants. This is likely the result of the micelle-microencapsulated, amorphous, and non-crystalline morphology and the use of natural emulsifiers and surfactants in the LMB formulation.

LMB, a lipid-based micelle delivery system, not only reached peak blood concentrations faster but could also sustain significantly higher berberine concentrations in participants over the study period (AUC_0–24h_) when using the same dosage of berberine. This suggests that when berberine is micro-encapsulated in a LipoMicel, a lower therapeutic dose can be employed, which may lead to fewer side effects (such as gastrointestinal ones related to the ingestion of large quantities of alkaloids) and higher patient compliance [63]. The formulation used in this research appeared to be safe; no side effects were reported throughout the study period. The strengths of this study include the presentation of in vitro results (i.e., in Caco-2 cell cultures) alongside human clinical data from a crossover study.

Crossover designs have several advantages for bioavailability studies as they remove inter-subject variability and have high power and statistical efficiency even with a smaller number of subjects [93]. One limitation associated with crossover designs is the carry-over effects. Therefore, in this study, a minimum washout period of 7 days has been included, which is in accordance with previous pharmacokinetic studies on berberine [34,77]. Limitations may include the small sample size of this pilot study (*n* = 10), as well as the non-randomization design. Also, since pharmacokinetic analyses were performed using a non-compartmental model, the data could not capture the complexities of berberine distribution in tissues and its eventual elimination. Future studies using the compartmental model could greatly improve our understanding of the underlying mechanisms that explain the observed differences.

Furthermore, since berberine and its derivatives display several pharmacological effects through various mechanisms, future clinical trials should evaluate possible mechanisms of actions of formulated products, in this case, LMB, which increase the blood concentrations of the alkaloid. The relationship between enhanced bioavailability and bio-efficacy (e.g., blood glucose- and lipid-lowering effects) could also be explored in future studies.

## 5. Conclusions

This study presents the absorption characteristics of a new food-grade berberine delivery system LMB (LipoMicel Berberine) in in vitro conditions, and a human pharmacokinetic study. The findings present that LMB has higher aqueous solubility and Caco-2 cell permeability compared to that of standard berberine, as well as up to six-fold greater absorption in our study participants over a 24 h period following the oral administration of 500 mg berberine. LMB reached approx. 10 times higher peak blood concentrations of berberine than the standard product and could sustain significantly greater blood uptake in participants over the study period (AUC_0–24h_).

Having an earlier maximum absorption and sustained higher blood concentrations could be meaningful for berberine supplementation in terms of its clinical efficacy. These results need to be tested in a larger human clinical study over a longer duration.

## Figures and Tables

**Table 1:** Study treatments.

Treatment	WBX	LMB
Dosage form	Hard-gelatin capsules	Soft-gelatin capsules
Berberine per capsule (mg)	500	250
Number of capsules per dose	1	2
Physical form of capsule content	Powder	Liquid
Non-medicinal ingredients	Carbohydrate gum [cellulose], purified water), microcrystalline cellulose, magnesium stearate (vegetable grade), stearic acid, silica.	gelatin, glycerin, purified water, carob powder, medium-chain triglycerides (coconut), bergamot flavor, msm, xylitol, stevia rebaudiana leaf extract, phosphatidylcholine lecithin (sunflower).

WBX: WellBetX (standard berberine); LMB: LipoMicel Berberine (delivery system of berberine).

**Table 2:** Pharmacokinetics of berberine formulations.

Product	AUC_0–24_ (ng h/mL)	C_max_ (ng/mL)	T_max_ (h)	K_el_ (h^−1^)	Initial Concentration (ng/mL)
WBX	13.4 ± 1.97	1.67 ± 0.41	5.60 ± 0.94	0.31 ± 0.1	0.58 ± 0.14
LMB	78.2 ± 14.4	15.8 ± 2.6	1.06 ± 0.21	0.09 ± 0.03	2.68 ± 1.72
*p*-value	0.0053	0.0041	0.0223	0.3783	0.8815

LMB: LipoMicel Berberine; WBX: WellBetX (standard berberine); AUC: the area under the blood concentration curve from the time of administration to 24 h; C_max_: maximum blood concentration; T_max_: time to reach C_max_; K_el_: elimination rate constant; *n* = 10; results are expressed as mean ± S.E.M.; *p* < 0.05 (ANOVA).

**Table 3:** Pharmacokinetics of female and male participants within the individual treatments.

	Male				Female			
Product	AUC_0–24_ (ng h/mL)	C_max_ (ng/mL)	T_max_ (h)	K_el_ (h^−1^)	AUC_0–24_ (ng h/mL)	C_max_ (ng/mL)	T_max_ (h)	K_el_ (h^−1^)
WBX	13.38 ± 2.80 ^a^	1.23 ± 0.27 ^a^	3.60 ± 1.50 ^a^	0.19 ± 0.1 ^a^	13.20 ± 3.25 ^a^	2.10 ± 0.76 ^a^	7.20 ± 1.20 ^a^	0.42 ± 0.16 ^a^
LMB	75.19 ± 16.9 ^b^	13.27 ± 4.17 ^b^	1.00 ± 0.35 ^a^	0.1 ± 0.04 ^a^	56.00 ± 9.73 ^b^	16.75 ± 0.82 ^b^	1.12 ± 0.32 ^b^	0.09 ± 0.03 ^a^
*p*-value	0.0397	0.0072	0.1120	0.7965	0.0227	0.0028	0.0018	0.0936

AUC: the area under the blood concentration curve from the time of administration to 24 h; Cmax: maximum blood concentration; Tmax: time to reach Cmax; K_el_: elimination rate constant; *n* = 5 (male); *n* = 5 (female); significant differences between pairwise comparisons between males and females indicated by different superscript letters “a, b” (*p* < 0.05, ANOVA); significantly different AUC_0–24_ was observed between the two treatments (LMB: LipoMicel Berberine; WBX: WellBetX (standard berberine)) for both genders; no significance was observed between the two genders.

**Table 4:** Solubility of berberine in different pH conditions.

Product	Water (mg/mL)	Simulated Intestinal Solution (mg/mL)	Simulated Gastric Solution (mg/mL)
LMB	2.34 ± 0.23	4.04 ± 0.28	0.44 ± 0.01
WBX	1.65 ± 0.01	5.42 ± 0.37	0.02 ± 0.01
*p*-value	0.0349	0.0210	0.0005

LMB: LipoMicel Berberine; WBX: WellBetX (standard berberine). Solubility evaluated in distilled water at pH 7.0; simulated intestinal solution at pH 6.8 and simulated gastric solution at pH 1.2; *p* < 0.05 and *p* < 0.001 (unpaired *t*-test).

**Table 5:** Apparent permeability coefficient (*P_app_*) values of berberine formulations.

Product	*P_app_* (cm/s) (±SEM)
LMB	7.18 × 10^−5^ ± 7.89 × 10^−6^
WBX	4.93 × 10^−6^ ± 4.28 × 10^−7^
*p*-value	<0.0001

LMB: LipoMicel Berberine; WBX: WellBetX (standard berberine); *n* = 8; *p* < 0.001 (unpaired *t*-test).

## Data Availability

Data and/or statistical analyses are available upon request.
